# The social contagion of temporal discounting in small social networks

**DOI:** 10.1186/s41235-020-00249-y

**Published:** 2021-03-03

**Authors:** Michael T. Bixter, Christian C. Luhmann

**Affiliations:** 1 Department of Psychology, Montclair State University, Montclair, NJ 07043 USA; 2 Stony Brook University, Stony Brook, NY USA

**Keywords:** Temporal discounting, Decision making, Small groups, Social influence, Social contagion, Direct influence, Indirect influence

## Abstract

Decisions often require a tradeoff between immediate and long-term gratification. How individuals resolve such tradeoffs reflects constructs such as temporal discounting, the degree that individuals devalue delayed rewards. Recent research has started to focus on temporal decisions made in collaborative contexts (e.g., dyads, small groups). Results suggest that directly interacting with others leads to revisions in preferences, such that decision makers become more similar to their collaborative partners over time (e.g., more patient following collaboration with a patient other). What remains to be seen is whether this social influence extends to indirect social effects, such as when an individual influences another’s preferences through a shared collaborative partner. In the current study, the focus was on decisions regarding hypothetical monetary rewards. Groups of three participated in a collaborative decision-making chain, in which network member X collaborated with member Y, who then subsequently collaborated with member Z. Though network members X and Z never directly interacted, a significant indirect link was observed between member X’s pre-collaborative decision preferences and member Z’s post-collaborative decision preferences. These results demonstrate that temporal decision preferences can be transmitted through intervening connections in a small social network (i.e., social contagion), showing that indirect social influence can be empirically observed and measured in controlled environments.

**Supplementary information:**

**Supplementary information** accompanies this paper at 10.1186/s41235-020-00249-y.

## Introduction

Many beneficial outcomes require a tradeoff between short-term and long-term gratification (e.g., good health, retirement savings). Resolving such tradeoffs reflects psychological constructs such as temporal discounting—the extent to which an individual prefers more immediate rewards over delayed rewards. Temporal discounting relates to many consequential behaviors, including addiction (Bickel et al. 2007), health outcomes (Amlung et al. 2016), and suicidality (Dombrovski et al. 2011). Therefore, a better understanding of how temporal preferences are formed and modified will have practical benefits for a variety of behavioral outcomes, particularly for interventions that target high rates of temporal discounting (e.g., Dennhardt et al. 2015).

Less is known about the degree to which temporal decision preferences are socially influenced. Humans exist within social networks, with their behaviors and preferences influenced by their social contacts (referred to as social contagion; Christakis and Fowler 2009). Social contagion has been observed in variables that involve the tradeoff between short-term and long-term consequences, such as smoking behavior (Christakis and Fowler 2008) and body-mass index (Christakis and Fowler 2007). Taken together, it might be expected that temporal decision preferences themselves exhibit strong social influence effects. However, most studies that looked at network effects have been observational studies, which make it difficult to isolate causal social influence. The current study aimed to help fill this gap in the literature by studying social influence on temporal decision making in a controlled environment. First, it is important to distinguish between two types of social effects: direct and indirect influence.

### Direct social influence

Direct social influence in a social network refers to the influence of one individual on another individual with whom they come into direct contact. Common examples include friends, family members, and work colleagues. Direct influence can stem from behavioral imitation, informational effects, and conformity to group norms. As it relates to temporal decision making, most research has focused on individual effects, making the role of social influence less clear. Recent research has demonstrated that observing the choices of a peer over the computer influenced intertemporal choices in a sample of young adults (Gilman et al. 2014). For example, observing an impulsive peer led to a greater preference for smaller-sooner monetary rewards over larger-later rewards.

Another way to test direct social influence is to have a group of participants interact together in a laboratory setting (Schweke et al. 2017; Tsuruta and Inukai 2018). In the collaborative decision-making paradigm (Bixter and Rogers 2019; Bixter et al. 2017), dyads or small groups of participants make decisions together during a collaboration phase. Because participants also complete the decision-making task individually both pre-collaboratively and post-collaboratively, direct social influence can be assessed by measuring the extent to which an individual’s preferences are related to how their partner’s preferences change from pre-collaboration to post-collaboration. That is, does collaborating with another person lead to revisions in decision preferences? Results from this paradigm demonstrate that the temporal decision preferences of group members are revised to be more similar post-collaboratively than they were pre-collaboratively. The extent of this direct social influence depends on the relative status that someone has within the group, with lower-status members exhibiting larger social influence effects than higher-status members (Bixter and Luhmann 2020).

### Indirect social influence

The social contagion of behavior ultimately relies on the spread of behavior through social networks, including between individuals who never directly interact with one another. An example of an indirect link is if individual X and individual Z never interact with each other, but both interact with individual Y. Therefore, indirect social influence refers to the influence of one network member on another member with whom they do not come into direct contact. Though direct social influence has been observed and measured in the social psychological literature for nearly a century (Asch 1956; Sherif 1936), indirect social influence is a relatively recent topic of study and permits the results to be more easily scaled to the complex structures and characteristics of social networks.

Most research that has focused on social network effects has used observational methods (e.g., Aral and Nicolaides 2017). In certain situations, behavior is not just influenced by direct contacts, but also by contacts separated by two degrees or more of separation (i.e., indirect links; Cacioppo et al. 2009; Rosenquist et al. 2010). These results suggest that behaviors can spread throughout social networks similar to contagious diseases. Observational social network studies have not gone without criticism, though, mainly due to them having difficulty separating causal social influence from other effects such as homophily (Shalizi and Thomas 2011) and context effects (Cohen-Cole and Fletcher 2008). Homophily refers to the tendency of people to choose their network connections based on shared characteristics (also referred to as *selection effects*). For instance, if clusters of individuals in social networks form their ties based on shared characteristics (e.g., smoking status, exercise habits), similarities observed between proximal network members might not be due to social influence. Context effects refer to environmental variables that may affect individuals in close proximity to one another (and are, thus, more likely to be connected within the larger social network). An example would be if a new fast food restaurant opens up next to an office building, which then leads to many of the workers in the office building (who share many direct connections) seeing their body-mass index scores increase over time.

Controlling the environment in laboratory settings can better isolate indirect social influence and information cascades more generally (Çelen and Kariv 2004; Anderson and Holt 1997). One area that has received particular attention is the spread of generosity, cooperation, and other pro-social behaviors (e.g., Jordan et al. 2013; Liu et al. 2015; Rand et al. 2011; Tsvetkova and Macy 2014). The findings regarding social influence on altruistic behavior have been mixed, with some studies finding significant indirect social influence (Fowler and Christakis 2010) and others not (Suri and Watts 2011). These experimental studies demonstrate that studying social networks in controlled environments helps to make clear the scope of indirect influence on decision behavior. For instance, even though direct social influence may be observed in a decision domain, social influence may not extend to indirect connections (e.g., Liu et al. 2015). In the context of temporal decision making, no laboratory study to date has measured whether decision preferences can be transmitted *indirectly* throughout small social networks.

By separating the discussion of direct and indirect social influence, we are not claiming that these two types of influences are fundamentally separate phenomena. After all, indirect social influence is just an aggregation of constituent direct social effects—if an indirect social effect links members X and Z through a shared link with member Y, this indirect influence derives from direct effects between members X and Y and members Y and Z. However, as the research above demonstrated, results are mixed on the presence of indirect social influence in decision making. As a result, just because direct social influence has been observed in a domain of interest such as temporal decision making, it does not necessitate that this influence will extend to indirect links within a social network. The presence or absence of indirect social effects places boundaries on the impact that social influence is likely to have in real social networks, making it a prime target of behavioral research.

### Overview of current study

**Fig. 1:**
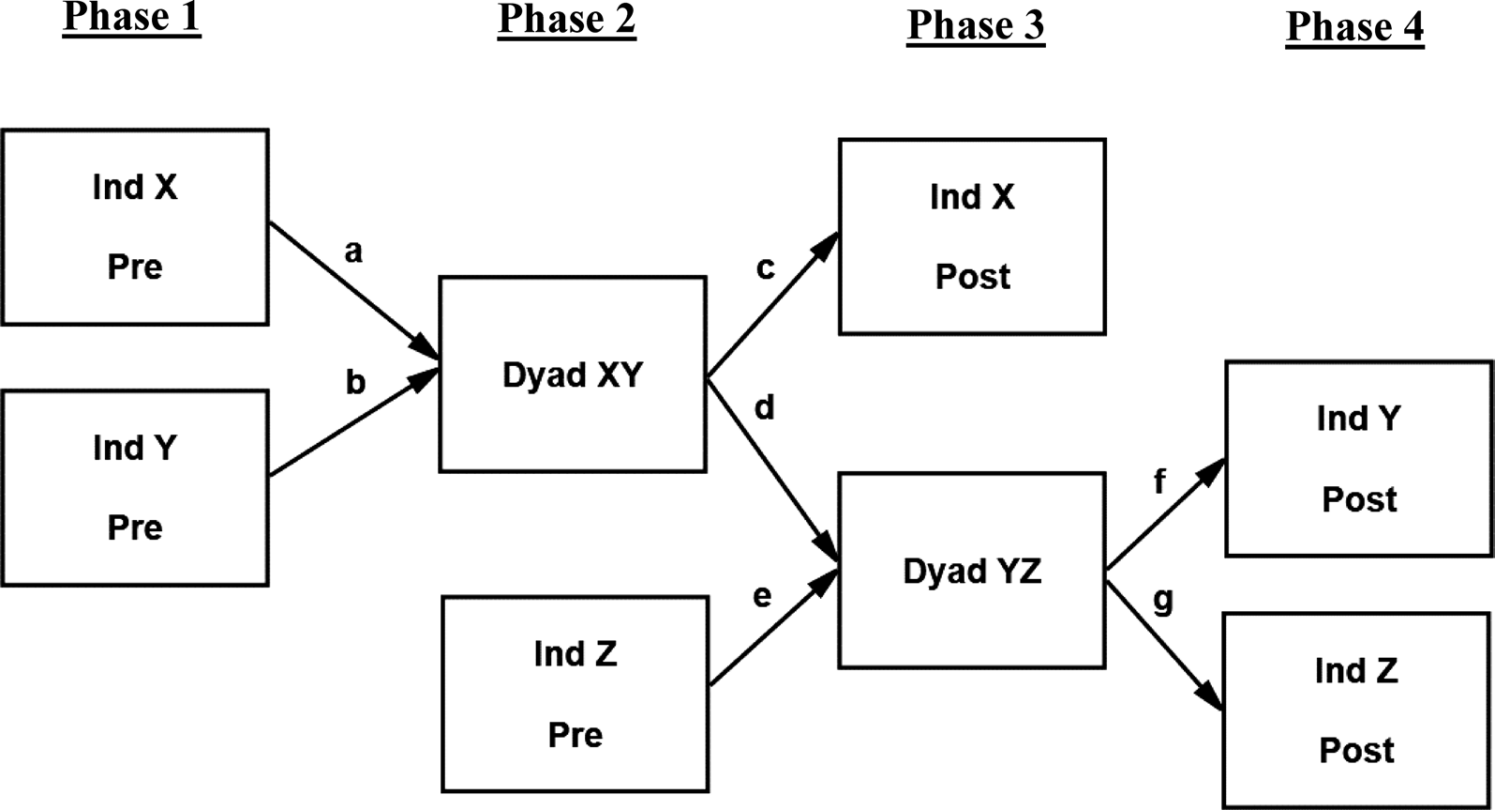
A visualiazation of the full path model fit to the small-network chain sequence of the current study. Three network members (X, Y, Z) completed a temporal decision-making task individually (Ind) and in collaborative dyads. The network members completed the individual conditions both prior to collaboration (Pre) and after collaboration (Post). The path lettering is used to help identify the various direct and indirect social effects included in Tables 1 and 2. Paths included in the model but not shown in the figure for illustration purposes include: direct paths from network members’ pre-collaborative discount rates to their respective post-collaborative discount rates (e.g., Ind X Pre → Ind X Post), covariances between network members’ pre-collaborative discount rates (e.g., Ind X Pre ↔ Ind Y Pre), and covariances between post-collaborative residuals (e.g., Ind X Post ↔ Ind Y Post). The latter covariances were included to take into account the dependency of the group data

To test both direct and indirect social influence on temporal decision making in a controlled, experimental environment, a sequential chain design was chosen. Small social networks consisting of three members (X, Y, Z) were included in each study session. All three network members first completed a temporal decision-making task individually to assess their baseline, pre-collaborative decision preferences regarding delayed rewards. Network members X and Y then collaborated together face-to-face as a dyad, where they completed the temporal decision-making task together. Subsequently, network members Y and Z collaborated together as a decision-making dyad. Finally, all three network members again completed the decision-making task individually to assess their post-collaborative decision preferences (see Fig. 1 for a visualization of the study sequence).

Due to the sequential chain structure of the study design, network members X and Z only shared an indirect link through participant Y. Therefore, any indirect relationship between member X’s pre-collaborative preferences and member Z’s post-collaborative preferences would demonstrate the social contagion of temporal decision making. Furthermore, due to the ordering of the sequence chain, influence between network members X and Z could only flow from X to Z, not vice versa. This study design allowed a more definitive test of indirect social influence compared to previous observational studies.

The focus on temporal decision making was due to intertemporal tradeoffs underlying many everyday decision conflicts. The particular task used in the current study dealt with hypothetical monetary rewards. Previous studies have often found no significant differences between temporal discounting tasks that used real vs. hypothetical monetary rewards (Johnson and Bickel 2002; Lagorio and Madden 2005; Madden et al. 2004). As a result, the use of hypothetical monetary rewards was deemed acceptable for this initial investigation of social network effects on temporal discounting. However, we describe in the “Discussion” section how future research can build upon the current study by utilizing a variety of incentivized and real-world decision tasks.

## Method

### Participants

Participants were 117 undergraduate students, consisting of 39 small social networks of three. The mean age of the sample was 19.7 years (SD = 2.40) and 54.7% were female. Participants received partial course credit for completing the study. The entire study took less than 1 h to complete.

### Materials

#### Temporal decision-making task

The decision-making task involved participants making decisions about hypothetical monetary rewards. On each trial, two rewards were presented on the screen. One of the two reward’s magnitude was missing (e.g., US$125 today or US$___ in 6 months). Participants’ task was to respond with the missing reward magnitude that would render them indifferent between the two rewards. Each implementation of the decision-making task consisted of 36 trials, with the order of the trials randomized. The 36 trials derived from three reward magnitudes (US$40, US$125, and US$250), three delays (3 months, 6 months, and 12 months), whether the sooner reward would be delivered today or also after a delay, and whether participants had to provide a missing value for the sooner reward or the later reward. The task was either completed individually or in a collaborative dyad, where the two network members would have to reach consensus on each trial.

#### Discount rates

Responses on each trial were converted to annual discount rates using Eq. 1 (Zauberman et al. 2009):

1 $${\text{Annual discount rate}} = { }\frac{{\left[ {\ln \left( {\frac{{{\text{X}}_{{{\text{t}} + {\text{h}}}} }}{{{\text{X}}_{{\text{t}}} }}} \right)} \right]}}{{\left[ {\frac{{\text{h}}}{12}} \right]}},$$

where *X*_*t*_ is the magnitude of the smaller-sooner reward, *X*_*t* + *h*_ is the magnitude of the larger-later reward, *t* is the delay associated with the smaller-sooner reward, and *h* is the additional delay (in months) associated with the larger-later reward. Higher discount rates imply greater devaluing of delayed rewards (i.e., greater impatience). The denominator involves dividing *h* by 12 to express the discount rate as an annual discount rate. For instance, if a participant replied US$150 in 6 months to the example trial above, the annual discount rate for that trial would be 0.36. However, if the participant required the reward to be US$500 in 6 months, the participant’s annual discount rate would be much higher at 2.77. Discount rates were calculated for each individual participant and each dyad by computing the discount rates implied by each response and then averaging the resulting set of discount rates across the 36 trials. The overall average discount rate across all network members and phases was 2.55 (SD = 1.03).

### Procedure

The design of the study can be seen in Fig. 1. Three participants (X, Y, Z) were included in a single study session as a small-network chain. Participants initially arrived and were placed into different testing rooms to minimize face-to-face interaction prior to any collaboration. Participants were not aware of the network design of the study or their location within the chain structure. The study was broken down into the following four phases (see Fig. 1).

- *Phase 1* Network members X and Y completed the temporal decision-making task as individuals (Ind X Pre and Ind Y Pre). They completed the task in separate rooms and were unaware that they would subsequently be completing a similar task in a collaborative context. These individual pre-collaborative tasks acted as baseline measures of the network members’ temporal decision preferences.
- *Phase 2* After completing their individual pre-collaborative tasks, members X and Y collaborated as a dyad (Dyad XY). During the collaborative task, the dyad members were asked to reach a consensual decision on each trial. As this collaborative task was being completed by members X and Y, the third member of the chain, participant Z, completed the task individually in a separate room (Ind Z Pre). Members X and Z never came into face-to-face contact to help to ensure that their indirect link was uncontaminated.
- *Phase 3* The first member of the chain, participant X, completed another individual phase of the task (Ind X Post). This phase acted as a post-collaborative measure of their temporal decision preferences following their direct interaction with participant Y. Also, during Phase 3, network members Y and Z collaborated together as a second dyad (Dyad YZ).
- *Phase 4* Network members Y and Z once again completed the temporal decision-making task individually (Ind Y Post, Ind Z Post). These individual tasks measured the two members’ post-collaborative decision preferences.

The sequential chain structure of the current study allowed both direct and indirect social influence to be measured within a single design and model. Direct effects could be assessed by the joint, multiplicative pathways between pre-collaborative and post-collaborative decision preferences of network members who directly interacted with one another. For example, did the pre-collaborative decision preferences of network member Y influence the post-collaborative decision preferences of member X following the Dyad XY experience? Moreover, because network members X and Z never directly interacted with one another, indirect social influence from X’s pre-collaborative decision preferences (Ind X Pre) to Z’s post-collaborative preferences (Ind Z Post) would demonstrate that temporal decision preferences can cascade and propagate throughout small social networks.

### Analysis plan

The path model displayed in Fig. 1 was fit to the data. This analytic approach allowed direct and indirect social influence to be estimated within a single model, as well as taking into account the dependency of the group data. Additional paths were estimated in the model but were not shown in Fig. 1 for illustration purposes, including direct paths from network members’ pre-collaborative decision making to their respective post-collaborative decision making (Ind X Pre → Ind X Post, Ind Y Pre → Ind Y Post, Ind Z Pre → Ind Z Post). These autoregressive path coefficients adjust network members’ post-collaborative discount rates by their respective pre-collaborative discount rates, so that any unique influences of the dyad interactions can be seen as influences on the change in network members’ discount rates. Covariances were also estimated between network members’ pre-collaborative decision making (Ind X Pre ↔ Ind Y Pre, Ind X Pre ↔ Ind Z Pre, Ind Y Pre ↔ Ind Z Pre), as well as covariances between the post-collaborative residuals (Ind X Post ↔ Ind Y Post, Ind X Post ↔ Ind Z Post, Ind Y Post ↔ Ind Z Post). These covariances were included to take into account the dependency of the group data.

The path models used maximum likelihood estimation with bootstrapped standard errors (based on 10,000 resamples). Bias-corrected 95% confidence intervals were used to test the significance of all path coefficients (including the indirect effects) of the models (MacKinnon et al. 2004); if the confidence limits of an interval did not include zero, the coefficient was deemed statistically significant.

## Results

Path coefficients derived from the path model in Fig. 1 are included in Table 1. We first report the various direct social effects in the model, followed by the indirect social effects. The multiplicative pathways derived from the path model that tested the various direct and indirect social influence effects are included in Table 2. Reported coefficients are the unstandardized estimates followed by the 95% bias-corrected confidence intervals in square brackets.

**Table 1:** Coefficient estimates of the path model displayed in Fig. 1

Dependent variable	Predictor	Path labeling (see Fig. 1)	Unstandardized coefficient (*B*)	Lower 95% CL	Upper 95% CL
Dyad XY (*R*^*2*^ = .59)					
	Ind X Pre	a	.30^a^	.104	.665
	Ind Y Pre	b	.56^a^	.339	.855
Ind X Post (*R*^*2*^ = .80)					
	Ind X Pre	–	.32^a^	.163	.591
	Dyad XY	c	.76^a^	.410	.972
Dyad YZ (*R*^*2*^ = .25)					
	Dyad XY	d	.35^a^	.080	.811
	Ind Z Pre	e	.21	-.021	.592
Ind Y Post (*R*^*2*^ = .81)					
	Ind Y Pre	–	.47^a^	.305	.748
		f	.64^a^	.473	.779
Ind Z Post (*R*^*2*^ = .78)					
	Ind Z Pre	–	.52^a^	.232	.800
	Dyad YZ	g	.65^a^	.282	1.008

Paths labeled as “–” were not included in Fig. 1 for illustration purposes. *CL* confidence limit. Confidence limits were derived from 95% bias-corrected bootstrap confidience intervals based on 10,000 resamples. The significance of the path coefficients (^a^) was based on whether the confidence interval included zero or not

**Table 2:** Direct and indirect social influence derived from the path model in Fig. 1

Dependent variable	Path	Path labeling (see Fig. 1)	Unstandardized coefficient (*B*)	Lower 95% CL	Upper 95% CL
Direct social influence					
Ind X Post					
	Ind Y Pre → Dyad XY	b × c	.43^a^	.179	.824
Ind Y Post					
	Ind X Pre → Dyad XY → Dyad YZ	a × d × f	.07^a^	.018	.161
	Ind Z Pre → Dyad YZ	e × f	.13	− .015	.388
Ind Z Post					
	Ind Y Pre → Dyad XY → Dyad YZ	b × d × g	.13^a^	.033	.318
Indirect social influence					
Ind Z Post					
	Ind X Pre → Dyad XY → Dyad YZ	a × d × g	.07^a^	.021	.187

*CL* confidence limit. Confidence limits derived from 95% bias-corrected bootstrap confidience intervals based on 10,000 resamples. The significance of the path coefficients (^a^) was based on whether the confidence interval included zero or not

### Direct social influence

The three-member chain structure of the current study allowed a variety of direct social effects to be estimated. The clearest test of a direct effect focuses on the first member of the chain, member X. This member only has a direct interaction with one other network member (member Y), and their individual decision preferences were assessed immediately before and after this collaboration.

The multiplicative pathway from member Y’s pre-collaborative discount rates (Ind Y Pre) to member X’s post-collaborative discount rates (Ind X Post), through the intervening dyadic collaboration (Dyad XY), was significant (*B* = 0.43 [0.179, 0.824]). This effect suggests that member Y exerted a significant direct social influence on member X’s revised preferences. That is, member Y’s baseline preferences had a unique effect on the decision preferences exhibited by the XY dyad during collaboration, which then uniquely predicted member X’s post-collaborative preferences. Because autoregressive paths were included in the path model (e.g., Ind X Pre → Ind X Post), any unique influence of the Dyad XY collaboration is on member X’s post-collaborative preferences adjusting for their baseline preferences.

To further investigate this direct social effect, a multiple regression was performed with member X and Y’s pre-collaborative discount rates (Ind X Pre, Ind Y Pre) predicting member X’s post-collaborative discount rates (Ind X Post). This model more clearly demonstrates the influence of member Y on member X by measuring the direct unique effect of member Y’s baseline preferences on member X’s revised, post-collaborative preferences. Both Ind X Pre (*B* = 0.55, SE = 0.11, *P* < 0.001) and Ind Y Pre (*B* = 0.42, *SE* = 0.13, *P* = 0.003) were significant unique predictors of Ind X Post, *F*(2, 36) = 21.63, *P* < 0.001, *R*^2^ = 0.55. These results demonstrate that network member X’s preferences were revised following the collaboration with member Y, so that their post-collaborative discount rates were a combination of their baseline preferences and the preferences of their collaborative partner. Specifically, interacting with a member Y who had higher (or lower) discount rates was associated with member X’s post-collaborative discount rates being higher (or lower), adjusting for their pre-collaborative discount rates.

Table 2 also includes other evidence of direct social influence. These influences include the multiplicative pathway from member X’s pre-collaborative preferences to member Y’s post-collaborative preferences (*B* = 0.07 [0.018, 0.161]) and member Y’s pre-collaborative preferences to member Z’s post-collaborative preferences (*B* = 0.13 [0.033, 0.318]). The pathway from member Z’s pre-collaborative preferences to member Y’s post-collaborative preferences did not quite reach statistical significance based on the bias-corrected confidence interval including zero (*B* = 0.13 [− 0.015, 0.388]). Though these above effects derive from joint, multiplicative pathways in the model, they are all still considered direct social effects because they trace the social influence between network members who interacted directly at some point during the chain sequence.

### Indirect social influence

The indirect social effect of interest was the influence of network member X on member Z, due to them never directly interacting during the study. The joint, multiplicative pathway between member X’s pre-collaborative discount rates and member Z’s post-collaborative discount rates was significant (*B* = 0.07 [0.021, 0.187]). That is, network member X’s preferences uniquely predicted Dyad XY’s decision making, which uniquely predicted Dyad YZ’s decision making, which then uniquely predicted member Z’s post-collaborative decision making. Similar to above, because autoregressive paths were included in the model (i.e., Ind Z Pre → Ind Z Post), these social influence effects on network member Z’s post-collaborative decision preferences are after adjusting for member Z’s baseline preferences.

**Fig. 2:**
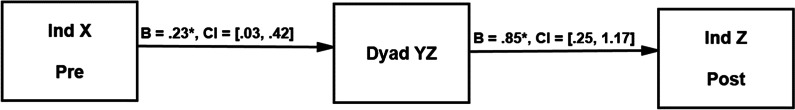
The reduced path model that measured the indirect social influence of network member X on network member Z through their shared link with member Y. Member X’s pre-collaborative discount rates (Ind X Pre) significantly predicted Dyad YZ’s discount rates even though member X was not directly involved in that collaboration. The significance of the path coefficients (*) was based on whether the 95% bias-corrected bootstrap confidience intervals included zero or not

To further test the indirect influence of network member X on network member Z, a reduced path model was fit to the data (see Fig. 2). In this model, a path was included between member X’s pre-collaborative discount rates (Ind X Pre) and Dyad YZ’s discount rates, and a path between Dyad YZ and member Z’s post-collaborative discount rates (Ind Z Post). The significant path between Ind X Pre and Dyad YZ (*B* = 0.23 [0.034, 0.419]) more clearly demonstrates the social contagion of decision preferences due to member X not being directly involved in the Dyad YZ collaboration phase. The joint multiplicative effect of these two pathways was also significant (*B* = 0.19 [0.058, 0.432]), again indicating a significant indirect social influence of network member X on network member Z.

## Discussion

The current study utilized small social networks to measure both direct and indirect social influence on temporal discounting in a controlled, laboratory setting. Participants’ post-collaborative discount rates were predicted by direct social interaction with another dyad member, supporting previous research (Bixter and Rogers 2019; Bixter et al. 2017). These results mean that collaborating with a high temporal discounter was associated with a network member exhibiting higher discount rates post-collaboratively, even adjusting for the network member’s baseline discount rates. The additional, novel finding of the current study is the presence of indirect social influence on temporal discounting. This was exhibited by a significant pathway connecting the decision preferences of two network members that did not share a direct link or interaction with one another. These results demonstrate that temporal discounting can be socially transmitted through intervening network members (i.e., social contagion).

How can decision preferences, such as temporal discounting, propagate throughout a social network? One explanation derives from prior research that showed a convergence effect in group temporal decision making, in which participants’ preferences are more similar following direct social interaction (Bixter and Rogers 2019; Bixter et al. 2017). This effect suggests that participants revise their preferences to be partially aligned with the preferences of their collaborative partner. As a result, when an individual then goes on to collaborate with a new network member, they are not solely bringing their own baseline preferences to the collaboration, but instead bring a mix of their own preferences and the preferences of the original network member. In the context of the current study design (see Fig. 1), after member Y collaborates with member X, member Y enters the second dyad collaboration (Dyad YZ) with preferences that are revised to be partially aligned with member X’s preferences. Insofar as member Z revises their own preferences based on the interaction with member Y, there still exists a trace of member X’s preferences in the behavior that is observed during the Dyad YZ collaboration. The results of the reduced path model shown in Fig. 2 most clearly demonstrate this idea. Member X’s baseline discount rates predicted Dyad YZ’s discount rates, even though member X is not directly involved in that latter dyad. Presumably, network member Y’s behavioral influence on Dyad YZ included the previous influence of collaborating with member X.

Prior research on the social contagion of behavior often utilized observational study methods (e.g., Aral and Nicolaides 2017). Studies that deal with pre-existing social networks in uncontrolled environments have difficulty controlling for homophily and context effects. Both of these latter phenomena can provide alternative explanations for social contagion effects (Cohen-Cole and Fletcher 2008; de la Haye et al. 2011). Studying structured social networks in laboratory environments help to isolate the presence of social influence effects. In these controlled environments, indirect social influence is sometimes not observed in decision-making behavior (e.g., Suri and Watts 2011). In the present study, focusing on decisions about delayed rewards, indirect social influence was observed and could be better attributed to social contagion. This is because control was placed on the order of the social interactions to ensure paths of influence could only flow in one direction. Specifically, network members X and Y first interacted with one another, followed by members Y and Z. Effort was made to ensure that there was no direct interaction between members X and Z. Therefore, any indirect effect between X and Z’s decision preferences can be better attributed to the transmission of preferences through member Y.

Future research will need to test the size, scope, and generalizability of the social contagion of decision making. For instance, observational network studies have observed social influence up to three degrees of separation (Christakis and Fowler 2007; Rosenquist et al. 2011). Laboratory studies can extend the current findings to measure the distance that indirect social influence can spread through small social networks in controlled environments. Moreover, it will be necessary for future research to study social network effects on decisions that more accurately model the everyday decisions that people encounter. For instance, the task used in the current study dealt with hypothetical monetary rewards. Though previous studies have often found no significant differences between individual discounting tasks that used real vs. hypothetical rewards (Johnson and Bickel 2002; Madden et al. 2003; Matusiewicz et al. 2013), and some group temporal decision-making studies have used financially incentivized tasks (Tsuruta and Inukai 2018), future social network studies need to incorporate decisions that involve real consequences. These results will help to establish the practical impact of direct and indirect social influence on decision making and related behaviors. In addition to incentivized tasks, it will be helpful to study social network effects on more realistic decisions that involve a tradeoff between immediate and delayed gratification. Instead of repeated trials dealing with two arbitrary monetary rewards, tasks can involve decisions in a simulated market environment that involve consumption and savings. How consumer preferences spread among direct and indirect network links will provide a more complete picture of peer influences on everyday decision making.

The overarching goal of the current study was to investigate the presence of social network effects on temporal decision making in a structured environment that controls for other extraneous factors. Now that social influence (both direct and indirect) has been shown to shape temporal decision preferences, it will be important for future research to focus on what variables impact the magnitude of this influence. For example, do different network structures produce more social influence and greater changes in temporal discounting? Does the degree of relationship between network members (e.g., close friends vs. strangers) moderate the magnitude of social influence? Once the focus is on assessing the magnitude of the change in temporal discounting, it will be helpful to have a no-social interaction control group as a baseline comparison. Any observed changes in temporal discounting can then be contrasted with any naturalistic changes that may occur in decision preferences. The present study did not include a control group because it was an initial investigation into whether indirect social influence can be observed at all in small social networks. Now that social network effects are established in the temporal decision-making domain, the next step will be to test what structures or manipulations amplify this social influence compared to a baseline.

Another area of future research is to see whether positive vs. negative behaviors propagate throughout social networks at different rates or magnitudes. Both positive behaviors (generosity; Fowler and Christakis 2010) and negative behaviors (e.g., drug use in adolescents; Ali et al. 2011) demonstrate social network effects. Yet, direct comparison of the spread of behaviors based on valence is limited (cf., Dimant 2019). The judgment and decision-making literature is well suited for this and related research questions, however. Experimental tasks can manipulate the valence of the decision domain, such as decisions regarding gains versus losses, and measure whether some decision preferences are more socially contagious than others. Findings of valence effects will be relevant for intervention researchers and public health practitioners, depending on whether the goal is to facilitate the spread of socially desirable behaviors or limiting the spread of maladaptive behaviors.

Due to individual differences in temporal discounting correlating with many consequential behaviors (e.g., addiction, obesity), recent studies have focused on measuring the impact of interventions on reducing high rates of discounting (Bickel et al. 2011; Dennhardt et al. 2015). Taking into account social network effects, some members may occupy more influential positions or nodes within local networks, making them desirable targets for behavioral intervention. In the laboratory, a recent study demonstrated the to which extent leaders and higher-status group members exert a stronger direct influence on others’ decision preferences (Bixter and Luhmann 2020). Future research should take into account network characteristics and dynamics to leverage social influence and optimize intervention effectiveness.

## Supplementary information

**Additional file 1:** Supplementary material.

## Data Availability

All data and study materials are included as supplementary materials. These materials include the data (in .csv and .sav formats), the implementation of the path models using two software programs (AMOS, R), and the experimental task code (as a PsychoPy .py script).

## References

[CR1] Ali MM, Amialchuk A, Dwyer DS (2011). The social contagion effect of marijuana use among adolescents. PLoS ONE.

[CR2] Amlung M, Petker T, Jackson J, Balodis I, MacKillop J (2016). Steep discounting of delayed monetary and food rewards in obesity: A meta-analysis. Psychological Medicine.

[CR3] Anderson LR, Holt CA (1997). Information cascades in the laboratory. The American Economic Review.

[CR4] Aral S, Nicolaides C (2017). Exercise contagion in a global social network. Nature Communications.

[CR5] Asch SE (1956). Studies of independence and conformity: I. A minority of one against a unanimous majority. Psychological Monographs: General and Applied.

[CR6] Bickel WK, Miller ML, Yi R, Kowal BP, Lindquist DM, Pitcock JA (2007). Behavioral and neuroeconomics of drug addiction: Competing neural systems and temporal discounting processes. Drug and Alcohol Dependence.

[CR7] Bickel WK, Yi R, Landes RD, Hill PF, Baxter C (2011). Remember the future: Working memory training decreases delay discounting among stimulant addicts. Biological Psychiatry.

[CR8] Bixter MT, Luhmann CC (2020). Delay discounting in dyads and small groups: Group leadership, status information, and actor-partner interdependence. Journal of Experimental Social Psychology.

[CR9] Bixter MT, Rogers WA (2019). Age-related differences in delay discounting: Immediate reward, reward magnitude, and social influence. Journal of Behavioral Decision Making.

[CR10] Bixter MT, Trimber EM, Luhmann CC (2017). Are intertemporal preferences contagious? Evidence from collaborative decision making. Memory and Cognition.

[CR11] Cacioppo JT, Fowler JH, Christakis NA (2009). Alone in the crowd: The structure and spread of loneliness in a large social network. Journal of Personality and Social Psychology.

[CR12] Çelen B, Kariv S (2004). Distinguishing informational cascades from herd behavior in the laboratory. The American Economic Review.

[CR13] Christakis NA, Fowler JH (2007). The spread of obesity in a large social network over 32 years. The New England Journal of Medicine.

[CR14] Christakis NA, Fowler JH (2008). The collective dynamics of smoking in a large social network. The New England Journal of Medicine.

[CR15] Christakis N, Fowler J (2009). Connected: The surprising power of our social networks and how they shape our lives.

[CR16] Cohen-Cole E, Fletcher JM (2008). Is obesity contagious? Social networks vs. environmental factors in the obesity epidemic. Journal of Health Economics.

[CR17] de la Haye K, Robins G, Mohr P, Wilson C (2011). Homophily and contagion as explanations for weight similarities among adolescent friends. Journal of Adolescent Health.

[CR18] Dennhardt AA, Yurasek AM, Murphy JG (2015). Change in delay discounting and substance reward value following a brief alcohol and drug use intervention. Journal of the Experimental Analysis of Behavior.

[CR19] Dimant E (2019). Contagion of pro- and anti-social behavior among peers and the role of social proximity. Journal of Economic Psychology.

[CR20] Dombrovski AY, Szanto K, Siegle GJ, Wallace ML, Forman SD, Sahakian B, Reynolds CF, Clark L (2011). Lethal forethought: Delayed reward discounting differentiates high- and low-lethality suicide attempts in old age. Biological Psychiatry.

[CR21] Fowler JH, Christakis NA (2010). Cooperative behavior cascades in human social networks. Proceedings of the National Academy of Sciences.

[CR22] Gilman JM, Curran MT, Calderon V, Stoeckel LE, Evins AE (2014). Impulsive social influence increases impulsive choices on a temporal discounting task in young adults. PLoS ONE.

[CR23] Johnson MW, Bickel WK (2002). Within-subject comparison of real and hypothetical money rewards in delay discounting. Journal of the Experimental Analysis of Behavior.

[CR24] Jordan JJ, Rand DG, Arbesman S, Fowler JH, Christakis NA (2013). Contagion of cooperation in static and fluid social networks. PLoS ONE.

[CR25] Lagorio CH, Madden GJ (2005). Delay discounting of real and hypothetical rewards III: Steady-state assessments, forced-choice trials, and all real awards. Behavioural Processes.

[CR26] Liu P-P, Safin V, Yang B, Luhmann CC (2015). Direct and indirect influence of altruistic behavior in a social network. PLoS ONE.

[CR27] MacKinnon DP, Lockwood CM, Williams J (2004). Confidence limits for the indirect effect: Distribution of the product and resampling methods. Multivariate Behavioral Research.

[CR28] Madden GJ, Begotka AM, Raiff BR, Kastern LL (2003). Delay discounting of real and hypothetical rewards. Experimental and Clinical Psychopharmacology.

[CR29] Madden GJ, Raiff BR, Lagorio CH, Begotka AM, Mueller AM, Hehli D, Wegener AA (2004). Delay discounting of potentially real and hypothetical rewards: II. Between- and within-subject comparisons. Experimental and Clinical Psychopharmacology.

[CR30] Matusiewicz AK, Carter AE, Landes RD, Yi R (2013). Statistical equivalence and test-retest reliability of delay and probability discounting using real and hypothetical rewards. Behavioural Processes.

[CR31] Rand DG, Arbesman S, Christakis NA (2011). Dynamic social networks promote cooperation in experiments with humans. Proceedings of the National Academy of Sciences.

[CR32] Rosenquist JN, Fowler JH, Christakis NA (2011). Social network determinants of depression. Molecular Psychiatry.

[CR33] Rosenquist JN, Murabito J, Fowler JH, Christakis NA (2010). The spread of alcohol consumption behavior in a large social network. Annals of Internal Medicine.

[CR34] Schweke D, Dshemuchadse M, Vesper C, Bleichner MG, Scherbaum S (2017). Let’s decide together: Differences between individual and joint delay discounting. PLoS ONE.

[CR35] Shalizi CR, Thomas AC (2011). Homophily and contagion are generically confounded in observational social network studies. Sociological Methods & Research.

[CR36] Sherif M (1936). The psychology of social norms.

[CR37] Suri S, Watts DJ (2011). Cooperation and contagion in web-based, networked public goods experiments. PLoS ONE.

[CR38] Tsuruta M, Inukai K (2018). How are individual time preferences aggregated in groups? A laboratory experiment on intertemporal group decision-making. Frontiers in Applied Mathematics and Statistics.

[CR39] Tsvetkova M, Macy MW (2014). The social contagion of generosity. PLoS ONE.

[CR40] Zauberman G, Kim BK, Malkoc SA, Bettman JR (2009). Discounting time and time discounting: Subjective time perception and intertemporal preferences. Journal of Marketing Research.

