# Supplementary material for: The social contagion of temporal discounting in small social networks
Source: Cogn Res Princ Implic. 2021 Mar 3;6:13. doi: 10.1186/s41235-020-00249-y (PMC8861223; doi:10.1186/s41235-020-00249-y)
Supplement: Supplementary file 1 — Additional file 1: Supplementary material. [file 41235_2020_249_MOESM1_ESM.zip › README.docx]

The AMOS scripts and files are for the path models included in the manuscript. The data are included in the data.sav file.

The full model files implement the full path model included in Figure 1. These files include:

-*fullmodel.vb*: this is a syntax-based version of the model using VB code. The only thing that needs to be modified is to include the computer path of the data.sav file on your local computer. You just need to paste the path into the line of code that states “ENTER FILE PATH HERE”.

- *full model.amw*: a graphical version of the full path model. You would just need to load the data.sav file from the location on your local computer. We also include an *indirect effects_full model.SimpleEstimand* file that the *full model.amw* file uses to more clearly present the indirect effect results of the path model.

The reduced model files implement the reduced path model included in Figure 2. These files include:

-*reducedmodel.vb*: this is a syntax-based version of the reduced model using VB code. The only thing that needs to be modified is to include the computer path of the data.sav file on your local computer. You just need to paste the path into the line of code that states “ENTER FILE PATH HERE”.

- *reduced model.amw*: a graphic version of the reduced path model. You would just need to load the data.sav file from the location on your local computer. We also include an *indirect effects_reduced model.SimpleEstimand* file that the *reduced model.amw* file uses to more clearly present the indirect effect results of the reduced path model.

We also include an R script that contains *lavaan* syntax to perform the path models included in the manuscript. The script file is called *analysis script*. We also include a .csv version of the data file (data.csv) to easily import into R.

Finally, we include the experimental task code (*Experiment Task Script*) as a PsychoPy .py script.
